# WCTECGdb: A 12-Lead Electrocardiography Dataset Recorded Simultaneously with Raw Exploring Electrodes’ Potential Directly Referred to the Right Leg

**DOI:** 10.3390/s20113275

**Published:** 2020-06-08

**Authors:** Hossein Moeinzadeh, Joseph Assad, Paolo Bifulco, Mario Cesarelli, Aiden O’Loughlin, Jonathan C. Tapson, Ibrahim M. Shugman, Aravinda Thiagalingam, Gaetano D. Gargiulo

**Affiliations:** 1 The MARCS Institute, Western Sydney University, Milperra 2214, Australia; j.tapson@westernsydney.edu.au; 2 Department of Cardiology, Liverpool Hospital, Liverpool 2170, Australia; jg.assad@gmail.com; 3 Department of Electrical Engineering and Information Technology (DIETI), “Federico II” The University of Naples, 80100 Naples, Italy; pabifulc@unina.it (P.B.); cesarell@unina.it (M.C.); 4 School of Medicine, Western Sydney University, Campbelltown 2006, Australia; aiden.oloughlin@gmail.com; 5 Cardiology Department, Campbelltown Hospital, Campbelltown 2006, Australia; shugmano@hotmail.com; 6 School of Medicine, The University of Sydney, Sydney 2006, Australia; aravinda.thiagalingam@sydney.edu.au; 7 School of Engineering, Western Sydney University, Kingswood 2747, Australia; g.gargiulo@westernsydney.edu.au

**Keywords:** electrocardiography, Wilson’s Central Terminal, unipolar leads

## Abstract

With this paper we communicated the existence of a surface electrocardiography (ECG) recordings dataset, named WCTECGdb, that aside from the standard 12-lead signals includes the raw electrode biopotential for each of the nine exploring electrodes refereed directly to the right leg. This dataset, comprises of 540 ten second segments recorded from 92 patients at Campbelltown Hospital, NSW Australia, and is now available for download from the Physionet platform. The data included in the dataset confirm that the Wilson’s Central Terminal (WCT) has a relatively large amplitude (up to 247% of lead II) with standard ECG characteristics such as a *p*-wave and a *t*-wave, and is highly variable during the cardiac cycle. As further examples of application for our data, we assess: (1) the presence of a conductive pathway between the legs and the heart concluding that in some cases is electrically significant and (2) the initial assumption about the limbs potential stating the dominance of the left arm concluding that this is not always the case and that might requires case to case assessment.

## 1. Introduction

The surface electrocardiography (ECG) demonstrates the electrical activity of the heart as it spreads towards the surface of the human body. Its most common incarnation is referred as 12-lead ECG, and it is known as one of the most valuable non-invasive diagnostic tools for cardiac assessment [1,2]. The 12-lead ECG is composed of three limb leads; three augmented leads and six precordial leads. The three limb leads (also known as bipolar leads) are referred as Lead I, Lead II, and Lead III, and calculated by the potential difference of electrodes placed on right arm (RA), left arm (LA), and left leg (LL) [2]:

(1) I=LA−RAII=LL−RAIII=LL−LA

Leads augmented Vector Left (aVL), augmented Vector Right (aVR) and augmented Vector Foot (aVF) are referred to as augmented leads (also known as Goldberger leads) and are measured as the potential difference between each limb electrodes and the average of the other two limb potentials [3]:

(2) $$\mathrm{aVL}=\mathrm{LA}-\frac{1}{2}\left(\mathrm{RA}+\mathrm{LL}\right)\;\mathrm{aVR}=\mathrm{RA}-\frac{1}{2}\left(\mathrm{LA}+\mathrm{LL}\right)\;\mathrm{aVF}=\mathrm{LL}-\frac{1}{2}\left(\mathrm{RA}+\mathrm{LA}\right)$$

The six precordial leads (V1 to V6) are synthesized by calculating the difference potential between each of the six chest electrodes potential (UV1:UV6) and the virtual reference point, known as Wilson Central Terminal (WCT) [4]. This reference point is measured as the average potentials of the right arm, left arm, and left leg electrodes’ potential [4]:

(3) $$\mathrm{WCT}=\frac{\left(\mathrm{LA}+\mathrm{RA}+\mathrm{LL}\right)}{3}$$

(4) $$V_{1}:V_{6}=\;\mathrm{UV}1:\mathrm{UV}6-\mathrm{WCT}$$

**Figure 1:**
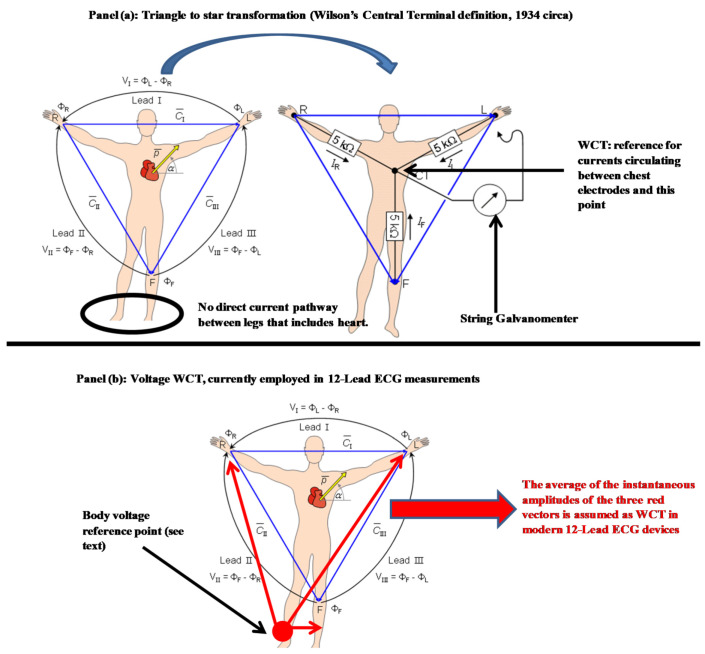
Traditional versus modern definition of Wilson Central Terminal (WCT; adopted from [5]).

While current measurements are considered absolute, voltage measurements are relative to a reference point. It is important to note that the original electrocardiography model was originally conceived using current measurements (string galvanometer). The original cardiac conduction model was rendered into a voltage model invoking the simplified homogeneous volume conductor hypothesis and the Ohm’s law, when the solid-state electronic amplifier made its debut in this field. For this reason, when considering the unipolar electrodes potentials in Equations (3) and (4) as they are supposedly measured to a common voltage reference this seems to cancel out. Unfortunately, in the case of the human body, where this reference point is directly or indirectly referred to the right leg, and the conductive pathways between exploring electrodes and this connection are not homogenous the cancellation of the reference point may not be perfect. This is particularly true for the WCT where potentials of chest electrodes, already referenced to right leg, are furtherly referenced to an average of electrodes (themselves referenced to the right leg) placed at a different distance from reference. As it is beyond the purpose of this paper to digress into the details of the model and the hardware utilized, we refer the reader to our previously published papers and in particular to [5] of which we adopted the conduction model depicted in Figure 1. In this paper, to simplify the notation we use the term unipolar lead to refer to the potential of Einthoven limbs’ electrodes (LA, RA, and LL) and the potential of the six electrodes on the chest (UV1:UV6).

Despite the 12-lead ECG has been used clinically for decades, there is still no mutual understanding of the WCT among researchers or cardiologists [6]. It is true that other more realistic cardiac conduction models have been proposed during the years, i.e., the Frank model [7] but these have not gained the traction required to enter clinical practice; exploring the reasons why these methods have not received the same research attention of the 12-lead ECG systems is beyond the scope of this brief communication. With this paper we would like to officially announce the availability of an ECG dataset available for download via the Physionet platform, under the name of WCTECGdb [8,9,10,11]. Although many ECG datasets with different features have been published [12,13,14], the WCTECGdb is a unique dataset as it contains the WCT signal and the unipolar leads. The dataset contains 540 ten seconds segments recorded from 92 volunteer patients. Each record comprises of signals for three limb leads, six precordials, three WCT components, six unipolar leads, and the WCT signal. In this paper, we demonstrate the characteristics of limb potentials, which lead to having a better understanding of the WCT signal. We also show that unipolar leads can be used to synthesize the traditional ECG leads. Together with the dataset we briefly discuss the potential clinical advantages of these new recordings together with some exemplificative applications of the data like the investigation over the relative amplitudes of unipolar limb leads to assess the presence of a conductive pathway between the legs and the heart as well as a comparison between the WCT potential and other leads.

## 2. Dataset Characterization

As mentioned, we have recorded data from patients at the Campbelltown hospital (New South Wales, Australia) over two years (2016–2018). All the patients volunteered for this study and gave written consent. This study was approved by the Ethics Committee of the South West Sydney Health District on 23 September 2015 with the protocol number HREC/15/LPOOL/302. The granted ethics clearance has been extended (it is still current, although all the non-necessary clinical trials have been suspended due to the COVID-19 outbreak) to increase the number of recordings.

We segmented each recording to ten second sections. Consequently, as the duration of the recording is different, each patient has a different number of segments, ranging between one to thirty-one. We recorded data from 92 patients (27 were female), and the total number of ten seconds segments is 540 for all patients. The average age of the patient population is 65.23 years (with a standard deviation of 12.12 years); patients had a history of cardiac disease and had been admitted to the hospital from the emergency department because of difficulties in breathing and/or chest pain.

This dataset comprises of raw and noise removed (cleaned) signals for the three limb leads, six precordial leads, nine unipolar leads including three WCT components, and six unipolar precordial leads. As the WCT is the average of the limb potential, we only added the cleaned WCT signal into the dataset. To pre-process our signals, we used the same filters employed for our other published studies. We employed a bandpass filter (0.05–149 Hz) and a powerline with harmonics up to the Nyquist frequency notch filter bank. All filters were implemented in MATLAB as 50th order IIR and applied with a zero-phase lag (bidirectional) [1].

In the uploaded dataset, we included supplementary information for each recorded segment such as the patient’s ‘age’, ‘gender’, ‘patient diagnosis’, and the ‘reconstructed precordials’ (if there is any). Each file in our dataset contained the signals and supplementary information listed in Table 1. Cleaned and raw signals were included in the dataset. The raw signals are specified by ‘-raw’ in the dataset (e.g., V2-raw) and refer to originally recorded signals prior to the noise filtering process. The WCT signal is only presented in a clean format.

The patient diagnosis as supplied in the dataset is presented in Table 2; unfortunately, the hospital could not provide us the diagnosis for ten patients. This is reflected in Table 2 under the patient diagnosis column as “not reported”.

We included synthesized precordial leads instead of directly measured signals for a total of 8 segments (from 5 patients), due to a poor signal to noise ratio and/or the final stage amplifier saturation. The signal saturation usually is seen when large contact impedances and electrode polarization generate large DC drifts at the unipolar potential that once amplified by the gain stages result in saturation of the output amplifier. As we recorded the potential of chest electrodes (UV1:UV6) and the WCT signal, we are able to reconstruct the missing precordial leads using Equation (4). It should be noted that both cleaned and raw data are reconstructed for these signals. We flagged these signals in the header file as “reconstructed precordials” and present the list of patients and signals in Table 3.

## 3. Lead’s Reconstruction

**Figure 2:**
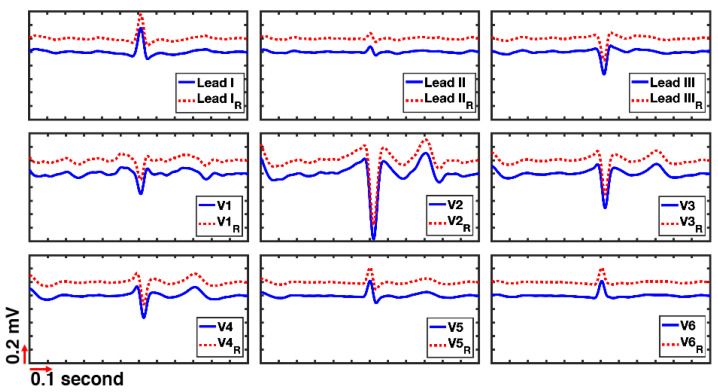
Comparison between recorded and reconstructed leads, shown with subscribed ‘R’. The reconstructed leads are shifted by 0.2 mV from the original place for better visualization (patient ID 6).

Each file contains two sets of recordings: (1) standard ECG leads including three limb leads (I, II, and III) and six precordial leads (V1:V6); (2) unipolar ECG leads comprising of three limb potential (LA, RA, and LL), six unipolar chest leads (UV1:UV6), and the WCT signal. It could be possible to reconstruct the standard ECG leads using unipolar leads by utilizing the Equations (1) and (4). Figure 2 illustrates the recorded leads in comparison with reconstructed (represented by subscribed ‘R’) ECG signals. The reconstructed leads are shifted by 0.2 mV from the original place to better presentation. Figure 2 shows that the synthesized and recorded leads are highly correlated and seem exactly the same.

We used the Sprague and Geers’ metric [15,16] to show the agreement between recorded and reconstructed signals. M_S&G_ provides the magnitude error, P_S&G_ gives the phase error, and C_S&G_ presents the overall error between recorded signals (*m*) and reconstructed signals (*p*) which both have a length of *N*. There is a more than 99% correlation between the reconstructed and recorded leads, which clarifies that the recorded and reconstructed signals are identical. The small differences (indistinguishable since that correlation for every lead exceeds 0.99) are due to components’ tolerances and mismatches. Table 4 presents the summary of the Sprague and Geers’ error and correlation between recorded and reconstructed leads for the 92 patients.

(5) $$M_{S\&G}=\sqrt{\overset{N}{\underset{i=1}{\sum }}p_{i}^{2}/\overset{N}{\underset{i=1}{\sum }}m_{i}^{2}}-1$$

(6) $$P_{S\&G}=\frac{1}{\pi }\;\cos^{-1}\overset{N}{\underset{i=1}{\sum }}p_{i}m_{i}/\sqrt{\overset{N}{\underset{i=1}{\sum }}m_{i}^{2}\;\overset{N}{\underset{i=1}{\sum }}p_{i\;}^{2}}$$

(7) $$C_{S\&G}=\sqrt{M_{S\&G}^{2}+P_{S\&G}^{2}}$$

## 4. Results and Discussion

**Figure 3:**
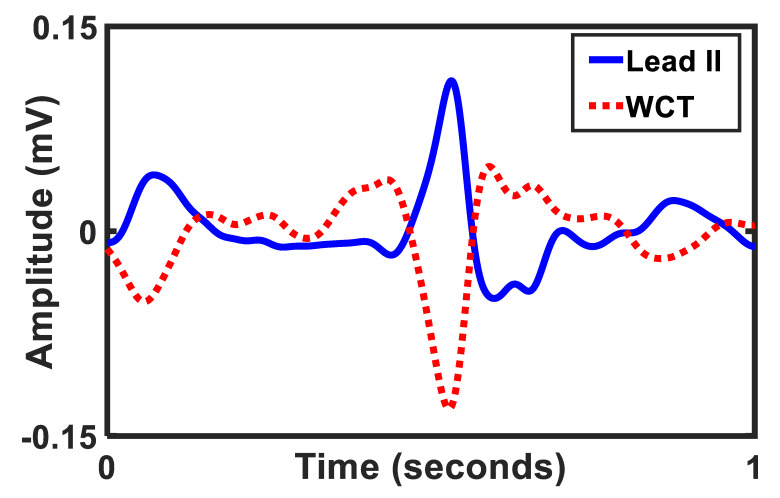
Example of the WCT and lead II amplitude (patient ID 32).

**Figure 4:**
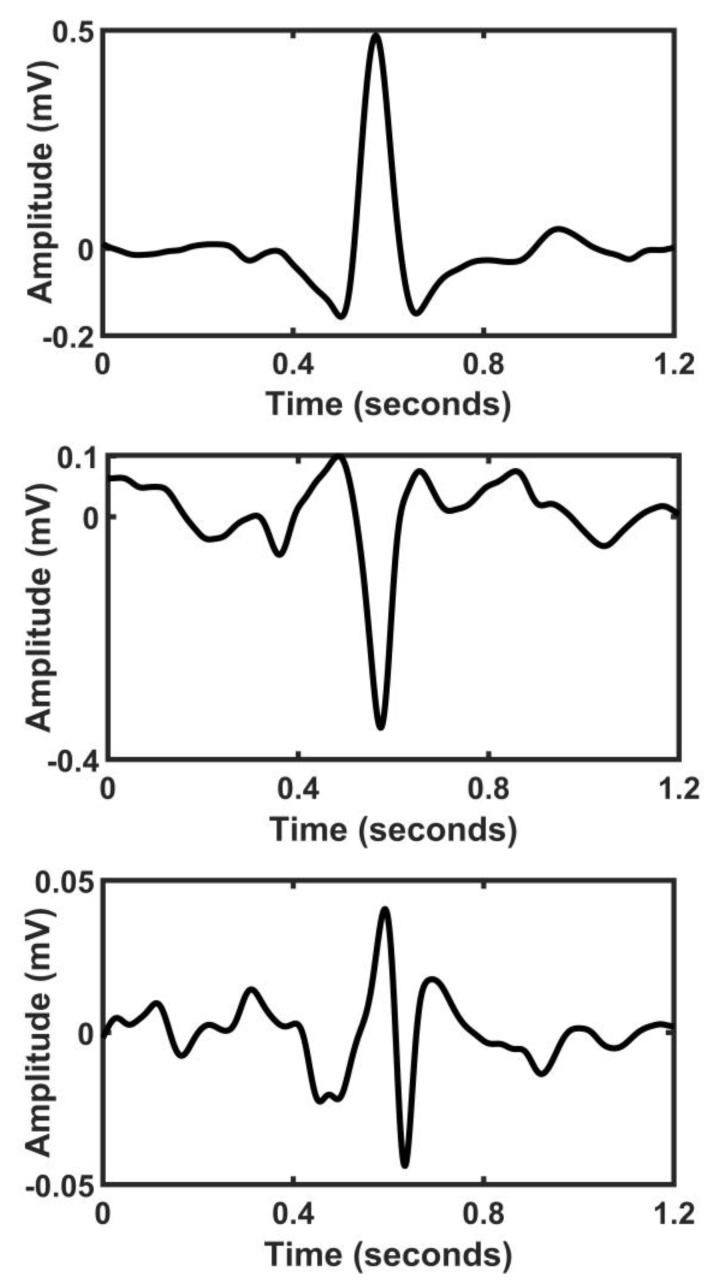
**Top** panel: the WCT with positive deflection (patient ID 44); **middle** panel: the WCT with negative deflection (patient ID 50); and **bottom** panel: the WCT with neutral deflection (patient ID 67).

We measured and reported the amplitude of the WCT as the percentage of lead II [5,17,18]. Our results support previous findings [19,20,21] to measure the WCT signal and showed that the WCT amplitude could be as high as 247% of lead II. Figure 3 is an example of a large WCT amplitude in relation to lead II. Furthermore, the WCT is highly individual, and it presents the standard ECG characteristics such as the *p*-wave and the *t*-wave. The distribution of the WCT polarity mostly has positive deflection, with some negative deflections and a handful of neutral polarities. We define as ‘neutral polarities’ those signals whose QRS as a bipolar mode of approximately equal positive and negative deflection [22]. Figure 4 demonstrates an example of positive, negatives, and neutral deflection of the WCT.

**Figure 5:**
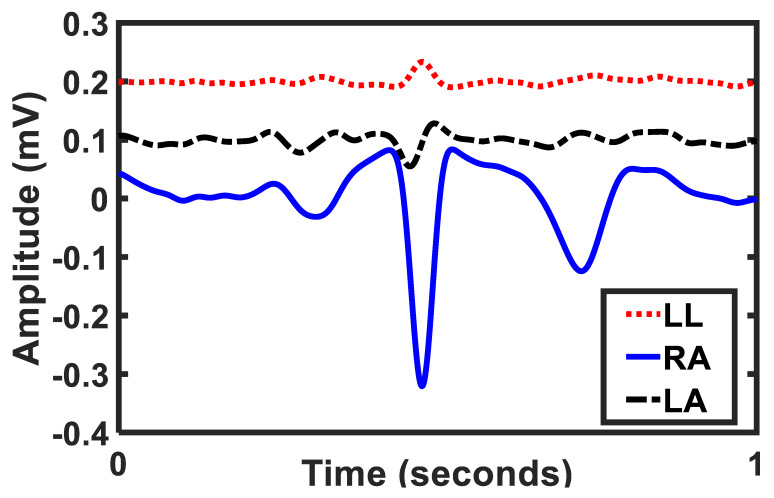
Comparison of the left arm (LA), right arm (RA), and left leg (LL) potentials. The RA has the peak to peak amplitude of 0.4 mV, while the LA has 0.07 mV, and the LL has 0.04 mV (patient ID 13).

This dataset provides the opportunity to have a better understanding of the limb potentials, as it contains the right arm, left arm, and left leg potential. The potential of limb electrodes depends on their location in relation to the heart. Since the left arm electrode is closer to the heart, it is assumed to have a larger amplitude while the left leg electrode has the largest distance from the heart and thus its potential amplitude assumed to be small or even negligible. However, our recordings show that this assumption was not correct for all patients. Figure 5 is an example of the patient with the right arm potential higher than the left arm and the left leg potentials. In this patient, the peak to peak amplitude of the left leg and the left arm were very close to each other, and in comparison, with the right arm potential, their amplitude was negligible.

**Figure 6:**
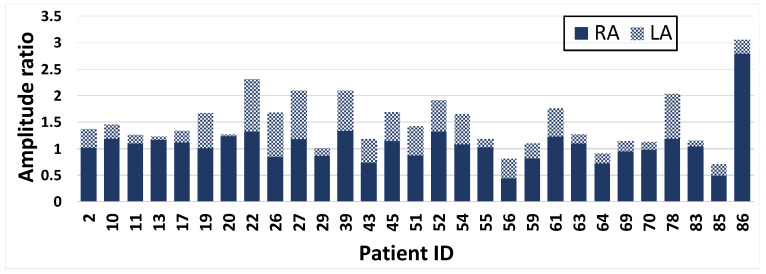
Patients with the RA potential higher than the LA potential. The average of three peak to peak amplitudes of the RA and the LA are measured respect with lead II.

**Figure 7:**
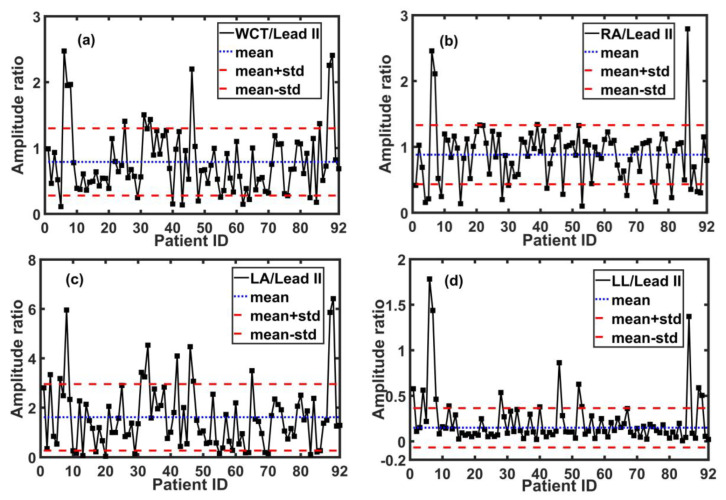
The amplitude of the WCT, LA, RA, and LL with respect to lead II for all 92 patients. Panel (**a**): the WCT/lead II is in the range of [0.11, 2.47] with an average of 0.78; Panel (**b**): the RA/lead II is in the range of [0.01, 2.7] with an average of 0.88; Panel (**c**): the LA/lead II is in the range of [0.038, 6.41] with an average of 1.61; and Panel (**d**): the LL/lead II is in the range of [0.007, 1.78] with an average of 0.22.

We measured the peak to peak amplitudes of limbs’ potential for three beats and reported the results in respect to lead II (Figure 6 and Figure 7). Our recordings show that the RA had a higher potential than the LA for 29 patients (Figure 6).

Furthermore, the left leg did not have a small amplitude for all patients (the LL/lead II was in the range of [0.007 mV, 1.78 mV] with an average of 0.22 mV); therefore, it could have a clinical influence on the WCT amplitude and the precordial leads for some patients. However, the average potential of the RA, LA, and LL with respect to lead II among all patients were 0.88, 1.61, and 0.22, respectively, which were aligned with the initial assumption. Figure 7 demonstrates the WCT and the Einthoven limbs’ potential amplitude with respect to lead II for all the 92 patients.

## 5. Conclusions

In this paper, we presented a unique dataset, which contained the WCT signal, six unipolar chest leads associated with three Einthoven limb leads, and six precordial leads. We recorded from 92 patients at Campbelltown Hospital (Campbelltown, Australia). We split each recording into ten second sections. Consequently, there were 540 segments in this dataset. Our recordings demonstrated that the WCT had ECG characteristics such as *p*-wave and *t*-wave. Additionally, our results confirmed the previous finding that the WCT is not a steady voltage reference during the cardiac cycle, thus, the WCT may be a new clinically relevant signal due to its high amplitude. We also presented the Einthoven limbs potential features for the first time. Our data undermined the initial assumption that the LL had a negligible amplitude and the LA had the highest potential among three Einthoven limbs. Furthermore, we showed that the 12-lead ECG signals could be synthesized using our unipolar leads (refereed directly to the right leg) with a high correlation (>0.99).

## 6. Appendix Further Notes on the WCT and Hardware 

As mentioned in the paper the WCT has been the object of debate since its inception and to date there is not a common understanding of what is WCT and where it is located.

What is the Wilson Central Terminal? The ideal reference point characterized by having (a) zero amplitude and (b) constant and steady in all places/times [23]. Only the infinity has these features, however, to find the feasible reference point respect to the volume conductor in the size of the human body, Wilson suggested using the central terminal [4,23]. As the electrical activity of the heart was assumed to be a single dipole in the centroid of the Einthoven triangle, he suggested averaging the potential of the limbs electrodes in order to estimate the potential of the dipole. Wilson assumed by using three large resistors connected to the limb electrodes, only a negligible current could pass. Therefore, each limb potential could be obtained. Nevertheless, Wilson’s theory initially absorbed researchers’ attention to work on measuring the real amplitude of the WCT [19,20,21,24,25,26,27,28]. The initial findings showed that the WCT amplitude could be as large as 40% of Einthoven’s ECG signals [19,21]. However, due to the difficulty of the WCT measurement, the method of measuring ECG using the WCT has been widely accepted and received scant research attention since the 1960s. 

**Figure 8:**
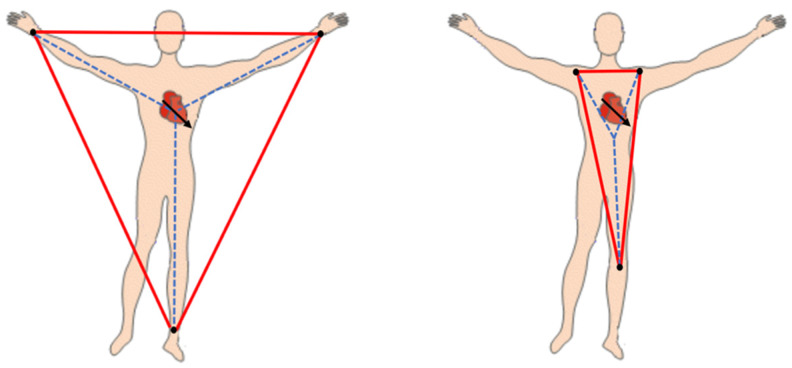
The measured WCT depends on the limb electrodes’ position. The WCT amplitude and location vary by changing the electrodes placed on the limbs.

Where is the Wilson Central Terminal? In theory, the WCT is formulated based on the simplified assumption that the electrical activity of the heart can be reduced to a single electrical dipole rotating around a fixed point in the chest [2,7] and located in the centre of the Einthoven’s triangle. This hypothesis is built upon the assumption that the geometrical positions of the limbs’ electrode construct the equilateral triangle, which is very unlikely in the routine ECG recording. In other words, the Einthoven triangle is not really an equilateral triangle, and the average potential of the limbs’ electrode cannot represent the assumed zero potential of the dipole (Figure 8).

What is the true unipolar chest leads? The precordial leads (V1:V6) have been referred to as unipolar leads in the literature, as the WCT is assumed having a “null” amplitude in the cardiac cycle. Contrary to the initial assumption, the WCT has a large amplitude and variability during the cardiac cycle, and the precordial leads are actually bipolar and should not be considered as “true unipolar”. Therefore, the WCT might remove important information from the potentials of chest electrodes. Furthermore, changing the position of electrodes on the limb causes the WCT amplitude to change and result in changing the precordial leads [29] (Figure 8).

In the past four years, we have developed and perfected (currently under trial) a new device, which enables recording of unipolar ECGs without using the WCT [22]. Our device indeed is a voltage recording device, as such it requires a reference point and a differential approach. Overall, our device uses a combined supply voltage bootstrap technique (to minimize the common mode noise) and a body reference placed to the right leg. According to the original inception of the surface electrocardiography postulated by Einthoven, as there is not a direct circuit that would make a current to flow between the legs that includes the heart directly in its pathway, we assumed that the right leg is the ideal reference point as it has the largest distance from the dipole (heart). Once can note that although there is no zero-potential in the human body, it is widely accepted that the right leg’s potential is near zero [30]. Therefore, we used this approximation to develop a new method to measure and store the WCT by recording directly from limb electrodes [1]. Our ECG device is able to record (1) the traditional ECG leads using the WCT as the reference point; and (2) the potential of electrodes on the limbs (RA, LA, and LL) and chest (UV1:UV6) using the right leg (RL) as the reference point. Therefore, we can measure the WCT amplitude, and the approximate unipolar leads, which are the raw biopotential measured from the exploring electrodes directly referred to the right leg (RL). The full hardware is fully described in [17].

## Figures and Tables

**Table 1:** The signal names and the detail of the recording presented for each segment.

**Signals**	I-raw	I	limb lead I
	II-raw	II	limb lead II
	III-raw	III	limb lead III
	V1-raw:V6-raw	V1:V6	precordial leads
	LA-raw, RA-raw, LL-raw	LA, RA, LL	three WCT components
	UV1-raw:UV6-raw	UV1:UV6	unipolar chest leads
		WCT	the WCT signal
**Detail**	Age Gender Patient diagnosis Reconstructed precordials *		

* Only included for 8 segments that needed to be synthesized for some of the precordial leads.

**Table 2:** Patient diagnosis list.

Patient Diagnosis	Count	Patient Diagnosis	Count
Angina	1	Non-ST segment elevation myocardial infarction (NSTEMI)	23
Atrial fibrillation	9	Pulmonary embolism-Atrial fibrillation	1
Atrial flutter	1	Pulmonary embolism	1
Atypical chest pain	5	Rapid atrial fibrillation with new cardiomyopathy	1
Cardiomyopathy	1	Rapid atrial fibrillation-pericarditis	1
Chest pain	1	Severe Mitral Stenosis	1
Complete Heart block	1	Sinus bradycardia	2
Congestive cardiac failure (CHF) exacerbation	1	Slow atrial fibrillation	1
Congestive cardiac failure (CCF)	1	ST segment elevation myocardial infarction (STEMI)	4
Coronary artery disease	3	Stable angina	7
Epigastric pain	1	Supraventricular tachycardia (SVT)	2
Fall secondary to alcohol intoxication	1	Syncope	3
Gastritis (non-cardiac chest pain)	1	Unstable angina	1
Hypertrophic obstructive cardiomyopathy	1	Urosepsis	1
Inferior ST segment elevation myocardial infarction (STEMI)	1	Ventricular tachycardia (VT)	3
Myocardial infarction-Type 2	1	Not reported	10

**Table 3:** List of patients with reconstructed precordial leads.

Patient ID	Segment ID	Reconstructed Precordial Leads
Patient7	Seg1	V2, V2-raw
	Seg2	V2, V2-raw
	Seg3	V1, V1-raw
Patient8	Seg1	V1, V2, V1-raw, V2-raw
	Seg2	V1, V2, V1-raw, V2-raw
Patient10	Seg1	V2, V2-raw
Patient14	Seg1	V2, V2-raw
Patient31	Seg1	V2, V2-raw

**Table 4:** The average Sprague and Geers’ error and correlation between recorded and reconstructed leads for 92 patients.

	Lead I	Lead II	Lead III	V1	V2	V3	V4	V5	V6
M_S&G_	0.012	0.013	0.023	0.017	0.026	0.020	0.019	0.018	0.023
P_S&G_	0	0	0	0	0	0	0	0	0
C_S&G_	0.012	0.013	0.023	0.017	0.026	0.020	0.019	0.018	0.023
Correlation	0.998	0.997	0.995	0.992	0.995	0.998	0.998	0.997	0.996

## Glossary

- Electrocardiography: bioelectrical recording from the heart. Abbreviated ECG and EKG. It contains three limb leads, three augmented leads, and six precordial leads.
- Reconstructed precordials: the difference between the potential of chest electrodes (UV1:UV6) and measured WCT potential using our new ECG device $V_{1}^{R}:V_{6}^{R}=UV1:UV6-WCT$. In this manuscript is referred to the chest leads, which are synthesized due to having difficulty in recording.
- Unipolar lead: voltage or potential of an electrode referred to the zero-reference point.
- Wilson’s Central Terminal: the imaginary reference point of the human body used to measure the precordial leads. Abbreviated as WCT. It is calculated by averaging the potential of right arm, left arm, and left leg, and has been assumed to have a zero amplitude.
